# 6th International Multithematic Scientific Bio-Medical Congress (IMBMC), Nicosia, Cyprus, 2018

**DOI:** 10.1038/s41419-019-1495-3

**Published:** 2019-03-15

**Authors:** Anthony Lisacek-Kiosoglous, Andrew Georgiou, Panayiota Christodoulou, Ioannis Patrikios

**Affiliations:** School of Medicine, European University Cyprus, Nicosia, Cyprus

The 6th International Multi-Thematic Scientific Bio-Medical Congress 2018 with the subtitle ‘Bio-medical Scientific Cyprus (BSC)’ was held at the European University Cyprus (EUC), Nicosia, Cyprus. EUC is established as an Institution with high-quality targets aiming to the excellence in academia, research, and innovation. BSC was founded and established by Professor Dr. Ioannis Patrikios, a faculty member of the School of Medicine at EUC; and is an annual event that has been internationally recognized.

The Key note speaker, Distinguished Professor Dr. Aaron Ciechanover (Nobel Laureate in Chemistry, 2004) gave the presentation entitled “Ubiquitin Proteolytic System—From Basic Mechanisms through Human Diseases and on to Drug Development.” The announcement of Professor Dr. Aaron Ciechanover as Honorary Professor of the School of Medicine, EUC was one of many highlights of this congress. The bestowal of the said honor was by the Rector of the EUC, Prof. Kostas Gouliamos.

Wolfgang Graier presented his research data about endoplasmic reticulum (ER) crosstalk as therapeutic targets against aging, cancer, and diabetes. He described how mitochondria are not only the masters of cellular energy metabolism but are involved in processes including signal transduction, biosynthesis, and gene expression. He described the recent attention on the ER–mitochondria axis, highlighting recent studies where inter-organellar Ca^2+^ transfer maybe a hallmark in diseases like alzheimers, diabetes mellitus, cancer, and ageing.

Aspasia Tsezou spoke on functional genomics and epigenetics in osteoarthritis (OA) and discussed evidence for metabolic deregulation. OA is characterized by progressive deterioration and loss of articular cartilage with concomitant structural and functional changes in the entire joint. Based on their research it was reported that OA chondrocytes have impaired expression of certain genes resulting in a reversal of cholesterol transport into the chondrocytes a critical step in the pathogenesis of OA. Furthermore, it was reported based on evidence that hsa-miR-33a regulates cholesterol synthesis and cholesterol efflux-related (ABCA1, ApoA1) genes and that it could serve as a potential serum biomarker for the evaluation of OA risk and progression and also as a potential novel target for the amelioration of the OA phenotype.

Achilleas Gravanis lectured on Neurogenic compounds as inducers of brain self-repair. Deficiencies in neurotrophins are implicated in the pathogenesis of many age-related neurodegenerative disorders, due to their central role in controlling adult neurogenesis. As he reported, the therapeutic applications of cognate polypeptide ligands for neurotrophin receptors are limited, and suggested that the development of nonpeptidergic, small-molecule ligands can overcome these limitations, and productively regulate this important receptor system with beneficial effects. He also discussed his current research on developing agonists of neurotrophin receptors (microneurotrophins) that induced fetal and adult neural stem cell self-renewal and differentiation, in vitro and in vivo; showing decreased hippocampal neurogenesis and deficient memory.

Alex Spyropoulos presented recent data regarding direct oral anticoagulants (DOACs) management, which suggest that any periprocedural management strategy should consist of an estimation of the procedural bleeding risk, patient renal function, and pharmacokinetic characteristic of each DOAC. He also presented consistent clinical data that points to the fact that the use of heparin-bridging therapy with DOACs lead to a multi-fold increased risk of major bleeding without an accompanying reduction in the risk of stroke or systemic embolism.

Giorgos Andrikopoulos gave a presentation entitled “New drugs, new tools for invasive treatment and new data from clinical trials on atrial fibrillation. Is there any hope for the treatment of the “incurable” arrhythmia?” Although antithrombotic therapy has successfully reduced the incidence of stroke and the development of novel anticoagulants (NOACS) expanded the use of proper antithrombotic therapy, the efficacy to prevent relapses of atrial fibrillation has been always very low due to the modest efficacy of antiarrhythmic medical therapy and its unfavorable influence on long-term prognosis of the patients, as he stated. He supplementary indicated that the introduction of invasive therapy of atrial fibrillation in the form of catheter and surgical ablation has substantially improved the antiarrhythmic strategies.

Adina T. Michael-Titus explained how a traumatic injury in the central nervous system can trigger a cascade of pathophysiological processes and that there is no neuroprotective or neurodegenerative treatment currently available. Furthermore, she discussed omega-3 fatty acids that have been shown in several models of central nervous system injury to have therapeutic and prophylactic potential. The link between neurotrauma and neurodegeneration were also discussed, and placed in the perspective of new concepts in the diagnosis and management of neurodegenerative conditions.

Philip C. Calder discussed how Omega-3 fatty acids have a role in prevention of coronary heart disease (CHD). He discussed the effects of omega-3 fatty acids with regard to cardiovascular diseases particularly CHD. He reported that the benefits of EPA and DHA were first identified in Inuit populations who had a very high intake but low mortality as a result of CHD, and presented various supporting studies. He further explained the protective mechanisms and beneficial impact of EPA and DHA on a range of risk factors for CHD exhibited in numerous individual trials and in a number of meta-analyses.

Nickolas Papadopoulos spoke about early detection of cancer and their vision of developing non-invasive tests. He pointed out that one of the main challenges in developing minimally invasive tests is the identification of the appropriate biofluid and cancer-specific biomarkers. He discussed the importance of DNA released from cancer cells and its use as a specific biomarker of cancer. He highlighted the use of PapSEEK test for the sensitive detection of endometrial and ovarian cancer using the Papanicolaou test liquid and the use of urine test called UroSEEK for the detection of bladder cancer.

Massimo Filippi presented a talk entitled “imaging the brain networks in demyelinating and neurodegenerative diseases”. He discussed the importance of MRI in the study of demyelinating and neurodegenerative diseases and he pointed out that even if other MRI measures (e.g. cortical lesions) may improve diagnostic specificity, they must have a standardized assessment and confirmed reliability. Novel MRI techniques increase the understanding of the disease and notably characterize the signature of each neurodegenerative condition, as he concluded.

Maddalena Lettino presented data on cardiovascular diseases (CVD), which are still the leading cause of death in European countries. She pointed out the mission of European Society of Cardiology (ESC), that is “to reduce the burden of cardiovascular disease” and the ESC-issued international guidelines on diagnosis and management of cardiovascular diseases.

Kyriakos Kypreos gave a talk about novel aspects of high-density lipoprotein (HDL) Biochemistry and Pharmacology. He stated that findings through their preclinical and clinical studies reinforce the idea that not all HDL particles are equally active and that apolipoprotein composition is a key factor for defining HDL lipid content and particle functionality.

Konstantinos Evangelou discussed the role of oncogene-induced senescence (OIS) in cancer. He explained the frequent p53 mutations in cancer and how apoptosis results in “clearance” of incipient cancer cells while escape from OIS, which is a viable state, remains an uncharted territory. He pointed out that detection of senescence is of paramount importance, especially in vivo, as it plays a bimodal role in cancer and seems to be related to prognosis. He concluded that recent findings have uncovered an unprecedented mode of how oncogenes drive cancer development, through escape from senescence, providing also new opportunities for cancer treatment.

Dimitrios Karussis spoke about stem cell therapies in neuroinflammatory and neuro-degenerative diseases. He explained the origin of stem cells and their function in the treatment of chronic degenerative and inflammatory neurological conditions and he discussed the pilot trials that performed in their centre using mesenchymal stem cells. He summarized the clinical experience with stem cells in neurological diseases, the promise, the hurdles and the risks of such experimental therapeutic approaches.

In this meeting report, we have summarized the major scientific findings from the aforementioned presenters and their respective topics. We note that unpublished data are also included.

